# Sir Arthur Stanley's Letter: The Status of Nurses

**Published:** 1918-02-16

**Authors:** A. Stanley

**Affiliations:** Chairman of the Council of the College of Nursing.


### Sir Arthur Stanley's Letter.

### The Status of Nurses.

To the Editor of "The Times."

Sir,—I fear I must trespass on your kindness to allow me a little space for a reply to Dr. Paterson's letter of the 21st instant, in order that I may support my contention that the differences between the Supplemental Charter of the Royal British Nurses' Association as agreed between that Association and the College of Nursing, and the same document as amended by the Privy Council, are immaterial.

Dr. Paterson contrasts the original Clause (*b*), which includes under the extended purposes of the Corporation "the promotion of a uniform curriculum and standard of qualification" with the amended Clause "the promotion of equivalent curricula and standards of qualification for all classes of Nurses." He omits to mention that the next Clause (*c*) of the Supplemental Charter, which was agreed between the Association and the College, about which there is no dispute, and in which the Privy Council made no alteration, expressly provides for the "institution and conduct of examinations and the grant of Diplomas and Certificates of Proficiency in Nursing or any branch of Nursing." All that the Privy Council, therefore, did as regards Clause (*b*) was to alter its phraseology the better to adapt it to Clause (*c*), since, if there are to be curricula and examinations and tests of proficiency in special branches of nursing, obviously there cannot be a uniform curriculum and a uniform standard, though there may be equivalent curricula, and equivalent standards of qualification, for all classes of nurses.

Dr. Paterson's second contention, that the omission by the Privy Council of the word "official" before the word "Register" in Clause (*d*) empowering the Corporation to "make and maintain an official Register of persons qualified to act as Nurses" is a substantial alteration, may be met by quoting the letter which the Clerk of the Privy Council wrote on this subject last June to the solicitors of the Royal British Nurses' Association. Sir Almeric Fitzroy remarks "In the Draft Supplemental Charter the use of the term may appear natural as applied to the Register of Nurses which is common to the two bodies proposed to be amalgamated." So it certainly appeared to us, and so the Council of the College of Nursing understood it. When, however, Sir Almeric continues "but in the language which the Royal Charter puts into the King's mouth the term can only suggest a State Register" we raised no objection whatever to the omission, since we never contemplated anything so disingenuous as by the use of the word official—quoting Sir Almeric again—"to anticipate the judgment of Parliament and prejudice the question of an official Register of Nurses." And greatly as we deplore the subsequent action of Dr. Paterson's Council in this matter, I do not believe that they themselves had any such thought at the time, particularly when I see that the very next Clause (*e*) of the Supplemental Charter specifically lays down a further purpose of the Corporation, the promotion of "legislation to provide for the State recognition of and protection of the official Register."

Once more, therefore, I feel myself justified in maintaining that what the Privy Council did when they desired us to omit the word "official" does not alter the plain and natural meaning of the Clause as it was agreed between the College and the Association, and moreover that the omission was immaterial, though fastened upon by Dr. Paterson and other members of the Royal British Nurses' Association as affording such justification as was possible for repudiating an agreement which had for other reasons become unacceptable.

With regard to the allegation that the altered draft of Clause (*b*), though communicated by the Privy Council to the solicitor of the Royal British Nurses' Association, was withheld from the knowledge of the Council of the Association "owing to the action of a member of the Council of the College of Nursing" from July of last year, it is quite true that there is one member only of the Council of the College of Nursing who at the date of the Privy Council's communication was an honorary officer of the R.B.N.A. For him to have acted in the way Dr. Paterson alleges may indeed afford evidence that in his view these alterations were of "some importance." For my own part, however, I find it difficult to believe the charge brought against this gentleman by his professional colleague whilst admiring the skill with which, "willing to wound and yet afraid to strike," Dr. Paterson seeks by innuendo to fix on the Council of the College of Nursing some part of the blame attaching to an action which, if done as alleged by a member of the

Council of the College of Nursing, was done solely in his capacity as an honorary officer of the Royal British Nurses' Association.—I am, Sir, etc.,

(Signed) A. STANLEY,

Chairman of the Council of the College of Nursing.

P.S.—To avoid possible misconception on the part of the public, I ought perhaps to add that the College of Nursing stands, as it has from the first, for the State Registration of Nurses, for a uniform curriculum, for a one-portal examination, and in the case of Nurses admitted to its Register after the expiration of the period of grace for not less than three years' training in a recognised hospital or infirmary.

